# Quality of professional life of intensive care unit workers during the COVID-19 pandemic

**DOI:** 10.47626/1679-4435-2023-1232

**Published:** 2024-11-14

**Authors:** Regina Claudia da Silva Souza, Mariana Davies Ribeiro Bersaneti, Ana Lúcia Siqueira Costa Calache

**Affiliations:** 1 Nursing School, Universidade de São Paulo, São Paulo, SP, Brazil; 2 Center for New Knowledge, Hospital Sírio Libanês, São Paulo, SP, Brazil

**Keywords:** COVID-19, compassion fatigue, health professionals, compassion satisfaction, burnout, covid-19, fadiga por compaixão, profissionais de saúde, satisfação por compaixão, burnout

## Abstract

**Introduction:**

Intensive care environment poses daily challenges to health workers. During the COVID-19 pandemic, intensive care unit staff experienced significant levels of secondary traumatic stress and burnout. Although they find satisfaction in their work, repeated exposure to the suffering caused by patients’ severe conditions exposes them to a high risk of compassion fatigue.

**Objectives:**

To evaluate the relationship between sociodemographic and professional variables and the professional quality of life represented by compassion satisfaction, secondary traumatic stress, and burnout during the first wave of the COVID-19 pandemic in a high-complexity hospital.

**Methods:**

This cross-sectional study was conducted from July to September 2020 in an intensive care unit of a Brazilian hospital. Participants were 89 health care workers who completed a questionnaire via the Research Electronic Data Capture platform, consisting of sociodemographic data and the Professional Quality of Life instrument. Professionals who were on leave for more than 2 months, enrolled in residency programs, or in leadership positions were excluded.

**Results:**

Levels of compassion satisfaction, burnout, and secondary traumatic stress were characterized as moderate, affecting 98.9%, 69.7%, and 59.5% of the sample, respectively. No sociodemographic variable was associated with professional quality of life.

**Conclusions:**

Workers showed moderate levels of compassion satisfaction, secondary traumatic stress, and burnout. No association was found between sociodemographic variables and professional quality of life. Understanding such issues among workers is crucial for the implementation of mental health promotion interventions.

## Abstract (pt)

**Introdução:**

O ambiente de cuidados intensivos apresenta desafios diários para os profissionais que atuam nessa área. Durante a pandemia de covid-19, esses desafios se intensificaram ainda mais. Embora esses profissionais obtenham satisfação com o trabalho, a exposição repetida a situações de sofrimento os coloca em alto risco de fadiga por compaixão.

**Objetivos:**

Avaliar a relação entre variáveis sociodemográficas e profissionais e a qualidade de vida profissional, representada por satisfação por compaixão, estresse traumático secundário e *burnout*, durante a primeira onda da pandemia covid-19 entre trabalhadores de unidade de terapia intensiva e analisar a correlação entre os fenômenos satisfação por compaixão, estresse traumático secundário e *burnout*.

**Métodos:**

Estudo transversal realizado de julho a setembro de 2020 em uma unidade de terapia intensiva de um hospital brasileiro. Participaram 89 profissionais, que responderam a um questionário com variáveis sociodemográficas e um instrumento de qualidade de vida profissional, enviado por meio da plataforma Research Electronic Date Capture. Foram excluídos profissionais afastados por mais de 2 meses, matriculados em programas de residência e os que ocupavam cargos de liderança.

**Resultados:**

Os níveis de satisfação por compaixão, *burnout* e estresse traumático secundário foram moderados, sendo identificados em 98,9%, 69,7% e 59,5% da amostra, respectivamente. Nenhuma variável sociodemográfica foi associada à qualidade de vida profissional.

**Conclusões:**

Os profissionais apresentaram níveis moderados de satisfação por compaixão, estresse traumático secundário e *burnout*, e as variáveis sociodemográficas não apresentaram correlação com a qualidade de vida profissional. O conhecimento desses fenômenos entre os profissionais é importante para implementar intervenções de promoção de saúde mental.

## INTRODUCTION

Professional quality of life (PQL) is the perception of an individual about their working conditions. Especially in health care, PQL encompasses two aspects: the positive (compassion satisfaction [CS]) and the negative (compassion fatigue [CF]) of caring for patients, especially those with severe illness.^1^ Intensive care environment poses daily challenges to workers in this field. During the COVID-19 pandemic, health workers who cared for critically ill patients and witnessed significant human suffering exhibited significant levels of secondary traumatic stress (ETS) and burnout.^2^ Although these workers derive professional satisfaction from their work, repeated exposure to suffering due to the severity of patient conditions puts them at high risk for CF.^2^ Intensive care unit (ICU) nurses are particularly at risk for burnout and CF,^3^ especially when faced with extreme situations such as terrorist attacks and environmental disasters.^4^

During the SARS-CoV-2 pandemic, with the increased risk of contamination and potential collapse of the health care system, professionals exposed to these conditions experienced feelings of insecurity and excessive tension that significantly affected their quality of life and work.^5^ Even highly skilled, resilient nurses experienced some degree of psychological distress, such as symptoms of posttraumatic stress and perceived stress.^5^ Major sources of stress included concerns about the lack and proper use of personal protective equipment, heavy workloads, fear of infection, and misinformation about the disease.^6^

Understanding the factors that may influence the QVP of ICU staff in extreme circumstances is critical since it directly affects their ability to provide care. A positive relationship has been observed between CS and caregiving ability, whereas a negative relationship has been observed between CF and caregiving ability.^7^

During the COVID-19 pandemic, moderate levels of perceived stress were observed among healthcare professionals, with 38% experiencing depression and 24% experiencing high levels of anxiety. ICU staff had higher levels of perceived stress than those working in other clinical areas.^8^ Since PQL is essential for effective human resource management and therefore for achieving good health outcomes, it is crucial to analyze it, especially in the context of a pandemic, when workers face increased workloads and greater risks to their safety. Many studies on this topic have been conducted in China and other countries.^9^

Therefore, the objective of this study was to evaluate the relationship between sociodemographic and professional variables and PQL, represented by CS, STS, and burnout, among health care workers during the first wave of the COVID-19 pandemic in a high-complexity private hospital in Brazil. In addition, the study analyzed the correlation between CS, STS, and burnout.

## METHODS

### STUDY DESIGN

This cross-sectional study was conducted from July to September 2020 in an ICU at a 490-bed private hospital in São Paulo, Brazil, treating patients with SARS-CoV-2 infection. The outcomes were level of CS, burnout and ETS among ICU healthcare workers and the relationship between sociodemographic and professional variables and PQL.

### STUDY SITE AND SAMPLE

The study was conducted in a 40-bed ICU with four subunits that served as a reference center for patients with COVID-19 during the pandemic. During the data collection period, the ICU had a 100% occupancy rate. Professionals worked 12-hour shifts every 36 hours. Social and psychological support programs were offered to all staff. The multidisciplinary team consisted of nurses, nursing technicians, physical therapists, physicians, nutritionists, pharmacists, and psychologists, for a total of 550 professionals. The convenience sample included 89 healthcare professionals, including nurses, pharmacists, physical therapists, physicians, nutritionists, and nursing technicians, who worked during the first phase of the pandemic. Workers on sick leave for more than two months, those enrolled in health residency programs, and those in leadership positions were excluded from the study.

### DATA COLLECTION PROCESS

An electronic survey was sent to workers three times between June and August 2020 via the Research Electronic Data Capture (REDCap) platform.^10^ Each study participant received the survey instrument via email. Upon completion of the survey, participant name was automatically removed from the list of workers. Questionnaire consisted of two parts: the first included sociodemographic data, and the second included the Professional Quality of Life 4 (ProQOL-4) instrument.^1^ Questionnaires were only available to participants after they had signed the informed consent form (ICF) provided on the same platform.

Outcome variables were levels of CS, STS, and burnout. Sociodemographic and occupational data regarding gender, age, marital status, presence of children, level of education, years of experience, professional category, salary range, weekly hours worked, and work shift were collected.

For the analysis, the ProQOL-4 scale was used to assess CS, STS and burnout. ProQOL-4 was developed by Stamm in 2005^1^ as well as adapted and validated for Brazilian Portuguese by Lago & Codo in 2010.^11^ In the present study, the adapted Brazilian short version was used,^11^ which has a Cronbach’s alpha of 0.81 for STS, 0.83 for burnout, and 0.76 for CS.^11^ The Brazilian version contains a total of 28 items.^11^

ProQOL-4 is a Likert scale with 28 items divided into three categories: CS, STS, and burnout. Individual item scores range from 1 (never) to 5 (very often). A score of up to 42 characterizes low levels, between 43 and 56 points moderate levels and above 57 points high levels of compassion satisfaction, burnout and secondary traumatic stress, according to the Stamm manual.^1^

### VARIABLES

Sociodemographic and professional characteristics were described according to the prevalence of the categories. Because of the small number of participants in categories other than nursing, the profession variable was divided into two categories: nursing staff (nurses and nursing technicians) and other health care workers (pharmacists, physical therapists, physicians, and nutritionists).

### ETHICAL CONSIDERATIONS

All participants selected for the study signed the ICF as well as were informed about the main aspects of the research and their right to refuse to participate or to withdraw from the study at any time. The study was conducted in accordance with the tenets of the Declaration of Helsinki (1964) and was approved by the Research Ethics Committee at the hospital, where was conducted, under opinion number 3.995.673.

### DATA ANALYSIS

Data extracted from the REDCap platform were organized and stored in an Excel Office 2010 spreadsheet. Normal distribution of the primary outcome variables was confirmed by using the Kolmogorov-Smirnov (KS) test, which allowed for the use of parametric tests. Means and standard deviations (SDs) were used for descriptive analysis. Analysis of variance (ANOVA) was used to compare the sociodemographic and professional variables with the mean T-score in the three subscales.

Scores obtained from ProQOL-4 were analyzed according to The Concise ProQOL Manual,^1^ prepared by the authors of the original instrument. The manual recommends converting data from the fourth to the fifth version by normalizing data and converting the raw scores to T-scores, which always have a mean of 50 and a SD = 10.^1^ All statistical analyses were performed using SPSS V20, Minitab 16, and Excel Office 2010. Correlations between subscale scores were calculated using Spearman’s test. Statistical significance was set at p < 0.05.

## RESULTS

The study included a sample of 89 health care workers, of whom 73 (82%) were women, 47 (52.8%) were between 34 and 51 years of age, 45 (50.6%) were nurses, and 66 (85.4%) worked up to 40 hours a week. The reliability of the instruments was verified using Cronbach’s alpha, with values of 0.71 for CS, 0.65 for STS, and 0.75 for burnout. ProQOL-4 scores showed 98.9% of the participants had a moderate level of CS, 69.7% had a moderate level of STS, and 59.5% had a moderate level of burnout (Table 1).

**Table 1:** Sociodemographic and professional characteristics of the health care staff in an ICU, São Paulo, 2020

Variables	n	%
Age range, years		
21 to 33	40	46.0
34 to 51	47	54.0
Sex		
Male	16	18.0
Female	73	82.0
Professional category		
Nursing staff	45	51.0
Other health care workers	43	49.0
Salary range, BMMS		
Up to 5.7	57	65.0
Above 5.7	31	35.0
Work shift		
Day	74	83.0
Night	15	17.0
Workload, hours		
30	28	31.4
36	37	41.5
40	11	12.3
More than 40	12	13.5
Marital status		
Without a partner	50	56.2
With a partner	39	43.8
Children		
Without	50	56.2
With	39	43.8
Level of education		
High school	14	16.0
Graduate studies	74	84.0
Number of jobs		
One	60	68.0
More than one	28	32.0
CS		
Low	01	1.1
Moderate	88	98.9
STS		
Low	24	27.0
Moderate	62	69.7
High	03	3.4
Burnout		
Low	36	40.5
Moderate	53	59.5

BMMS = Brazilian minimum monthly salary; CS = compassion satisfaction; ICU = intensive care unit; STS = secondary traumatic stress.

### BRAZILIAN MINIMUM MONTHLY SALARY (BMMS)

Median scores were 49.3 for CS, 49.2 for STS, and 49.9 for burnout (Table 2). According to the T-score classification, these scores correspond to moderate levels.

**Table 2:** T-scores for CS, STS, and burnout among health care workers, São Paulo, 2020

	Mean (SD)	Median	Q1	Q3	Min	Max
CS	50.0 (10.0)	49.3	44.7	56.1	17.2	67.6
STS	50.0 (10.0)	49.2	43.0	55.4	30.5	80.4
Burnout	50.0 (10.0)	49.9	44.0	55.9	32.2	73.6

CS = compassion satisfaction; SD = standard deviation; STS = secondary traumatic stress.

Sociodemographic and professional variables analyzed with the T-score results for CS, STS, and burnout did not show statistically significant differences (Table 3).

**Table 3:** Comparison between sociodemographic variables and ProQOL-4 T-scores, São Paulo, 2020

Variable	CS	p-value^*^	STS	p-value^*^	Burnout	p-value^*^
Age range, years						
21 to 33	49.6	0.683	49.47	0.926	49.59	0.888
34 to 51	50.47		49.66		49.89	
Sex						
Female	50.16	0.744	50.67	0.180	50.12	0.806
Male	49.25		46.95		49.44	
Professional category						
Nursing staff	50.22	0.744	50.06	0.998	48.97	0.307
Other health workers	49.52		50.06		51.17	
Salary range, BMMS						
Up to 5.7	50.18	0.987	50.73	0.309	50.52	0.495
Above 5.7	50.21		48.44		48.98	
Work shift						
Day	49.91	0.563	50.45	0.273	50.58	0.216
Night	51.55		47.32		47.04	
Marital status						
With a partner	50.60	0.618	50.71	0.555	50.84	0.485
Without a partner	49.53		49.44		49.34	
Children						
With	50.37	0.760	50.19	0.873	49.53	0.697
Without	49.71		49.85		50.37	
Level of education						
High school	50.56	0.864	46.96	0.224	47.12	0.240
Graduate studies	50.06		50.54		50.57	
Number of jobs						
One	50.36	0.684	48.70	0.062	48.82	0.157
More than one	49.42		52.98		52.05	
Weekly workload						
30	50.73	0.605	50.59	0.874	49.65	0.635
36	50.99		50.67		49.88	
40	46.54		48.20		48.68	
More than 40	49.25		49.06		53.55	

BMMS = Brazilian minimum monthly salary; CS = compassion satisfaction; ProQOL-4 = Professional Quality of Life 4; STS = secondary traumatic stress.

* Analysis of variance (ANOVA) test.

Burnout had a negative correlation with CS, and there was a positive correlation between burnout and STS. Both correlations were moderate (Table 4).

**Table 4:** Correlation between ProQOL-4 scores, São Paulo, 2020

	CS	STS
STS		
r	-0.172	
p-value^*^	0.108	
Burnout		
r	-0.403	0.544
p-value^*^	< 0.001	< 0.001

CS = compassion satisfaction; ProQOL-4 = Professional Quality of Life 4; STS = secondary traumatic stress.

* Spearman’s correlation.

Major causes of fatigue reported by health workers were fear of contamination (33.7%), conflict with the multidisciplinary staff (32.6%), work overload and long shifts (15%), and adverse clinical outcomes (10%). Major coping strategies were spiritual resources (40%), social support (15%), relaxation activities (10%), and psychological support (10%).

## DISCUSSION

During the SARS-CoV-2 pandemic, health care workers faced intense psychological pressure and work overload, leading to numerous studies analyzing the impact of this event on their lives, particularly on their PQL.^2,5,12^ These conditions highlighted the need for public policies including support, motivation, protection, training, and educational interventions to help workers cope with such significant challenges.^13^

In the present study, some personal and professional characteristics were analyzed in relation to PQL. Overall, the results provide evidence consistent with previous studies on the subject, indicating personal and professional characteristics did not influence levels of CS, STS, and burnout.^14,15^

The prevalence of the age group between 34 and 51 years and the predominance of women in this study are consistent with other studies conducted in 2020 with nurses in hospitals in Brazil and Spain, which analyzed the psychological impact of SARS-CoV-2 on nursing professionals.^12,16^ Marital status and number of children were the personal characteristics that differed, with a higher proportion of married workers and those with children in the Spanish study.^16^ Women showed a greater presence of stressors, higher emotional distress, and lower use of coping strategies, which may be related to their role in the family context.^14^ No sociodemographic variable in this study was associated with the topic studied. However, other studies have found different results, showing relationships between sex, occupation, weekly workload, and years of work experience.^17,18^

As in the study by Del Pozo-Herce et al.,^16^ participants in the present study were young. Age influences the perception of stressors, as the impact of these stressors decreasing with age. This may be related to work experience and adaptation to the work environment.^16^ Age should be considered when developing interventions including social and psychological support for health care workers, especially younger ones.

The absence of high levels of burnout in our sample is consistent with findings from a study conducted in Italy during the most critical phase of the first wave of the pandemic, where low levels of burnout were associated with moderate levels of CS.^14^ This situation probably occurred because various factors contribute to the compensation of CS in relation to CF during the pandemic. The analysis conducted during the first wave of the pandemic does not fully capture the true impact of the situation; in addition, there is a lack of information on the mental health of healthcare workers prior to the COVID-19 pandemic.^14,19-21^ Low levels of burnout were also observed among healthcare workers who were on the front lines during the first wave of the pandemic in China.^21^

Findings from the present study showed moderate levels of burnout, similar to those observed in a nursing staff in southern Brazil during the same period in 2020.^12^ The correlations between the three subscales were also similar in both studies, with CS showing a moderate inverse correlation with burnout and a weak correlation with STS.^12,15^

In the present study, there was a negative correlation between burnout and CS and a positive correlation between burnout and STS. Although both correlations can be classified as moderate, this finding is significant and consistent with what the literature supports on this topic.^12^

In the present study, the level of CS among health workers was moderate. Although they recognize the level of stress associated with their work, the work also provides significant rewards that can alleviate work-related stress and fatigue.^22^ Stress and anxiety levels are higher among nurses, while burnout is more prevalent among physicians.^22^ This may be explained by the fact that physicians and nurses are the health care workers who most often make clinical and care decisions.

Although this study did not find an association between personal and professional variables and the phenomena studied, the literature indicates that younger age, female gender, lack of social support, social isolation, stigmatization, working in a high-risk environment, professional category of nurse, low level of specialized training, and work experience are associated with a higher likelihood of developing mental health problems among health care workers.^23^ Severe emotional distress particularly affects critical care nurses.^24,25^

Fear of contamination, conflict with team and family, adverse clinical outcomes, severity of patients, and working conditions were highlighted by participants in this study. These stressors were confirmed by health care workers in a study conducted in eight European countries during the first wave of the pandemic in 2020, where participants reported that the most common stressors were uncertainty about the end of the COVID-19 pandemic and fear of infecting their families.^26^

Strategies related to spiritual resources and social support were most frequently mentioned by participants in the present study, confirming the perceptions of professionals in other countries regarding these resources.^27-29^ Other means, such as improving protective measures and gaining more knowledge about COVID-19, were also used by some health workers.^26^ Although there is limited evidence on the impact of these interventions in reducing mental health problems among health care workers during pandemics, their implementation is important to protect the mental health of health care workers.^30^ The risk factors identified in the studies are modifiable and represent important targets for future interventions.^30^

The present study had limitations such as the sample size, the cross-sectional design, and the lack of knowledge about the mental health of workers before the COVID-19 pandemic. These aspects make it difficult to compare the results, generalize the findings, and fully understand the phenomena.

## CONCLUSIONS

The sociodemographic variables showed no association with PQL. Burnout had a moderate inverse correlation with CS and a direct correlation with STS. The professionals had moderate levels of CS, burnout and STS. Future studies with a longitudinal design and evaluation of interventions to improve PQL would be valuable in understanding how to achieve better working conditions and mental health for professionals.
